# Comment on “Reduced temporal muscle thickness predicts shorter survival in patients undergoing chronic subdural haematoma drainage” by Korhonen et al.—The authors' reply

**DOI:** 10.1002/jcsm.13826

**Published:** 2025-05-07

**Authors:** Tommi K. Korhonen, Otso Arponen, Moritz Steinruecke, Ilaria Pecorella, Harry Mee, Stefan Yordanov, Edoardo Viaroli, Mathew R. Guilfoyle, Angelos Kolias, Ivan Timofeev, Peter Hutchinson, Adel Helmy

**Affiliations:** ^1^ Division of Neurosurgery Department of Clinical Neurosciences Cambridge University Hospitals NHS Foundation Trust & University of Cambridge Cambridge UK; ^2^ Department of Neurosurgery Neurocenter OYS Oulu University Hospital Oulu Oulu Finland; ^3^ Department of Neurosurgery Research Unit of Clinical Neurosciences University of Oulu Oulu Finland; ^4^ Department of Radiology University of Cambridge Cambridge UK; ^5^ Faculty of Medicine and Health Sciences Tampere University Tampere Tampere Finland; ^6^ Department of Radiology Tampere University Hospital Tampere Tampere Finland

We thank Du et al. for their comments [1] on our recent manuscript suggesting lower temporal muscle thickness (TMT) is a prognostic factor for shorter survival following surgical management of chronic subdural haematoma (CSDH) [2]. Du et al. raised several important remarks from our manuscript. First, they discuss the application of two‐ and three‐dimensional temporal muscle measurements in addition to muscle thickness only. Second, they review the breadth of laboratory and clinical biomarkers and their potential effects on outcomes following CSDH surgery. Third, they detail surgical techniques in relation to overall outcomes and conclude suggesting prospective studies to evaluate the benefit from intensified nutritional therapy.

While we share our colleagues' enthusiasm towards additional methods to measure temporal muscle dimensions perhaps in more detail, we wish to underline that TMT is currently the most established and researched method for the assessment of temporal muscle ‘mass’ [3, 4, 5, 6]. The lack of automated workflows currently renders the otherwise useful measurement of two‐ and three‐dimensional temporal muscle indices a cumbersome task. Comparative studies evaluating the optimal muscle index are currently unavailable. Based on the available literature, we regard TMT as the gold standard for temporal muscle evaluation.

We agree with Du et al. that in CSDH, as most other clinical conditions, many laboratory‐based, radiological and clinical markers in addition to muscle status may affect outcomes. These are often inadequately captured in studies utilizing traditional retrospective or prospective methodologies. With respect to causes of muscle mass loss, we believe clinical and laboratory markers related to generalized vulnerability, e.g., sarcopenia, cachexia, malnutrition and frailty are of interest [7]. Several relevant associations between laboratory biomarkers and TMT, most previously unreported, were removed from our manuscript [2] during its peer review process. We have submitted these supporting results as a separate manuscript.

Citing recent advancements in CSDH management, Du et al. highlight that relatively minor changes in surgical technique may significantly affect outcomes. For instance, irrigation of the subdural space has only recently been found beneficial compared to not irrigating [8]. A novel therapy, embolization of the middle meningeal artery, appears useful in preventing CSDH recurrences [9, 10, 11]. As accurately pointed out by Du et al., a subset of our patients had undergone a (mini)craniotomy instead of CSDH evacuation via burr holes, and most had received general anaesthesia instead of local. The surgical technique is chosen individually by the oncall consultant and the type of anaesthesia an institutional protocol. Although they may affect outcomes, neither of these parameters could be controlled in our retrospective study. This underlines the requirement of prospective research, as Du et al. suggest.

While nutritional status would be a compelling explanation for reduced imaging‐detected muscle mass, we propose a more nuanced prospective study, as low muscle mass is not a diagnostic criterion in the well‐established diagnostic criteria of malnutrition [12, 13]. Indeed, despite extensive evidence suggesting reduced muscle mass is associated with poor prognosis [14], the current understanding of the multifaceted causes of reduced muscle mass and its potential role in clinical work is severely limited. Improved understanding in this area is required to design appropriate studies to evaluate whether TMT holds predictive potential in addition to its prognostic role as suggested by Du et al. Such information is needed to set out evidence‐based recommendations for the management of patients with low imaging‐detected muscle mass.

## Conflicts of Interest

The authors declare no conflicts of interest.
